# Disparate movement behavior and feeding ecology in sympatric ecotypes of Atlantic cod

**DOI:** 10.1002/ece3.7939

**Published:** 2021-07-26

**Authors:** Martin Lykke Kristensen, Esben Moland Olsen, Even Moland, Halvor Knutsen, Peter Grønkjær, Anders Koed, Kristi Källo, Kim Aarestrup

**Affiliations:** ^1^ National Institute for Aquatic Resources Technical University of Denmark Silkeborg Denmark; ^2^ Institute of Marine Research Flødevigen Marine Research Station His Norway; ^3^ Centre for Coastal Research Department of Natural Sciences University of Agder Kristiansand Norway; ^4^ Aquatic Biology Department for Bioscience University of Aarhus Aarhus Denmark

**Keywords:** Atlantic cod, behavior, ecotypes, stable isotopes, telemetry, trophic ecology

## Abstract

Coexistence of ecotypes, genetically divergent population units, is a widespread phenomenon, potentially affecting ecosystem functioning and local food web stability. In coastal Skagerrak, Atlantic cod (*Gadus morhua*) occur as two such coexisting ecotypes. We applied a combination of acoustic telemetry, genotyping, and stable isotope analysis to 72 individuals to investigate movement ecology and food niche of putative local “Fjord” and putative oceanic “North Sea” ecotypes—thus named based on previous molecular studies. Genotyping and individual origin assignment suggested 41 individuals were Fjord and 31 were North Sea ecotypes. Both ecotypes were found throughout the fjord. Seven percent of Fjord ecotype individuals left the study system during the study while 42% of North Sea individuals left, potentially homing to natal spawning grounds. Home range sizes were similar for the two ecotypes but highly variable among individuals. Fjord ecotype cod had significantly higher δ^13^C and δ^15^N stable isotope values than North Sea ecotype cod, suggesting they exploited different food niches. The results suggest coexisting ecotypes may possess innate differences in feeding and movement ecologies and may thus fill different functional roles in marine ecosystems. This highlights the importance of conserving interconnected populations to ensure stable ecosystem functioning and food web structures.

## Graphical abstract

We investigated the behavior and trophic ecology of sympatric ecotypes of Atlantic cod. Differences in both behavior and trophic ecology were found between the ecotypes. This suggests coexisting ecotypes may possess innate differences in feeding and movement ecologies and may fill different functional roles in marine ecosystems, thus highlighting the importance of conserving interconnected populations to ensure stable ecosystem functioning and food web structures.

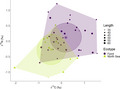

## 1 INTRODUCTION

The evolutionary divergence of ecotypes is common in both terrestrial and aquatic ecosystems and represents an important component of intraspecific diversity. A large body of empirical and theoretical studies have examined the evolution of ecotypes, for instance in the context of ecological speciation (Hendry, 2017). Ecotype variation may also have wide‐ranging consequences for ecosystems. For instance, anadromous salmon ecotypes support freshwater and terrestrial ecosystems by transporting large amounts of nutrients from oceanic ecosystems as part of their feeding and spawning migration (Carlson et al., 2011). Understanding potential eco‐evolutionary effects of ecotype variation is therefore highly relevant for conservation and management.

The Atlantic cod (*Gadus morhua*) is an iconic marine fish found across coastal and offshore shelf areas in the North Atlantic Ocean. Traditionally, a variety of morphs and life history forms have been recognized (Karlsen et al., 2013; Sherwood & Grabowski, 2010). Migratory forms in, for example, Northern Norway, Iceland, and Canada utilize shallow or coastal areas for spawning and open oceans for feeding while sedentary forms in, for example, Iceland, Canada, and Southern Norway are fjord residents during most of their lifecycle (Pálsson & Thorsteinsson, 2003; Wroblewski et al., 2005). Parallel to this, different color morphs may represent variants with overlapping distribution areas but different preferences in terms of food or habitat (Gosse & Wroblewski, 2004). In Skagerrak, southern Norway, two genetically differentiated ecotypes coexist within coastal habitats. Individuals assigned to the “North Sea” ecotype are genetically similar to cod sampled from offshore spawning grounds in the North Sea and most likely conform to this population, in contrast to assignments to local “Fjord” ecotype more commonly sampled from inshore coastal populations (Knutsen et al., 2018). This evolutionary divergence of the Fjord and North Sea ecotypes could in fact represent intermediate stages of an ecological speciation process (Roney et al., 2018). However, the genomic inversions that separate the two ecotypes (Sodeland et al., 2016) which might be both old and stable represent potential for persistent local adaptations and limited scope for subpopulation mixing (see Barth et al., 2019). Even within similar habitats such as eelgrass beds or kelp forests, the North Sea ecotype typically grows faster and reaches a larger juvenile body size compared to the fjord ecotype (Jørgensen et al., 2020; Knutsen et al., 2018), suggesting that they may have different ecological roles, including feeding and behavioral strategies. Also, there is empirical support for the North Sea ecotype having lower fitness (survival) in the fjord environment compared to the local fjord ecotype (Barth et al., 2019).

Cod is recognized as a cornerstone species and dominant top predator that may shape the trophic structure and function of marine ecosystems. When cod populations collapsed in Atlantic Canada, there was a correlated change in fish biodiversity affecting the stability of the entire ecosystem (Ellingsen et al., 2015). In coastal Skagerrak, the decline of cod has been linked to a trophic cascade leading to the degradation of nearshore seagrass and seaweed habitats (Östman et al., 2016). There could be a negative feedback loop on cod recruitment linked to this trophic cascade, since seaweed, and particularly the seagrass habitats, represent high‐quality nursery areas where juvenile cod have larger growth compared to more barren habitats (Knutsen et al., 2018). Cod fisheries in Skagerrak are highly diverse and involve a significant recreational fishery and commercial fishing (Fernández‐Chacón et al., 2017; Kleiven et al., 2016). Both fisheries mainly catch the North Sea ecotype, probably reflecting the Fjord ecotype being in a depleted state (Jorde, Kleiven, et al., 2018; Knutsen et al., 2018). Therefore, there is an urgent need to understand the ecological function of the fjord ecotype compared to the oceanic North Sea ecotype.

Here, we explore the detailed movement ecology and trophic role of the Fjord and North Sea ecotypes within a fjord system. To this end, we apply a novel combination of acoustic telemetry, population genetics, and stable isotope analyses. We hypothesize that the Fjord ecotype will display a more resident behavior in the inner parts of the fjord compared to the North Sea ecotype. Based on current knowledge about juvenile growth rates (Jørgensen et al., 2020), we also anticipate that the two ecotypes will have different trophic niches.

## 2 MATERIALS AND METHODS

### 2.1 Study area

**FIGURE 1:**
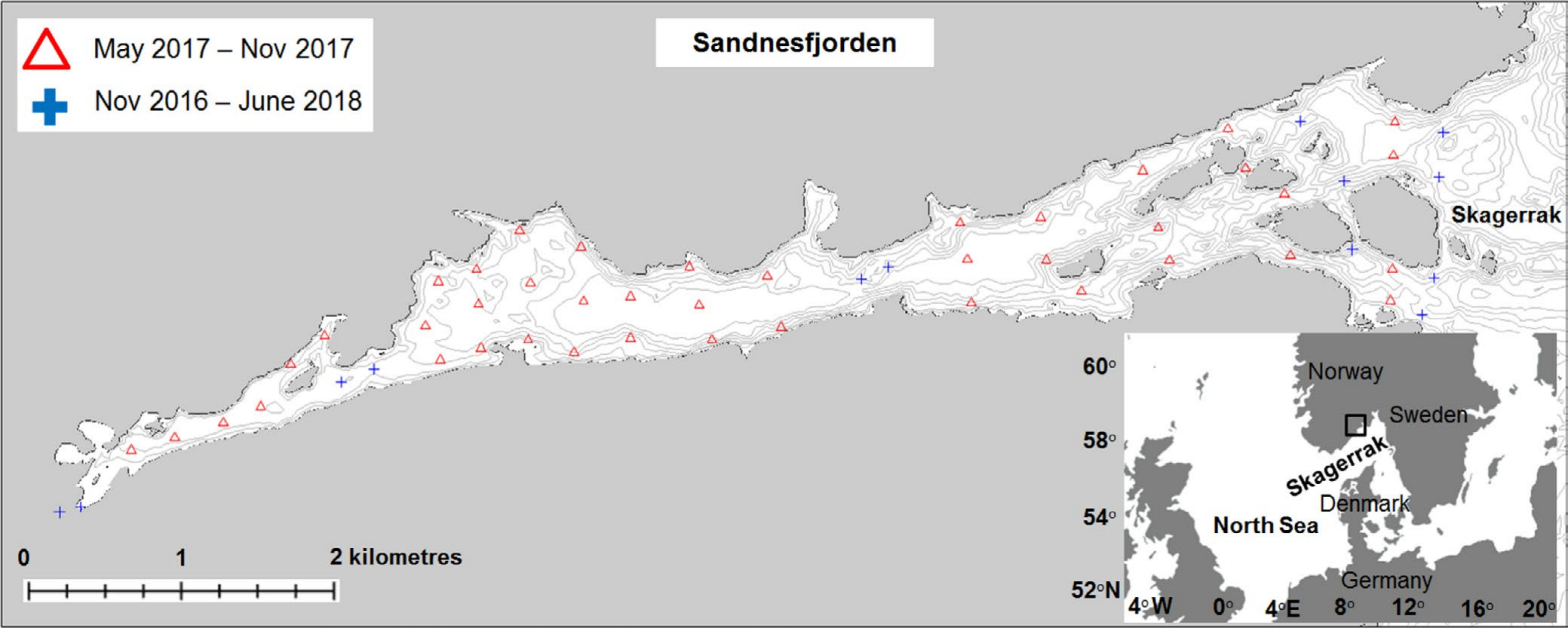
Map of the Sandnesfjord with red triangles representing positions of receivers in the array and blue crosses representing positions of receivers deployed throughout the study period

The study took place in the Sandnesfjord, a nine km long fjord system located on the southern coast of Norway (Figure 1). The Sandnesfjord is 70 m deep at the deepest point and has a mixture of bottom substrate types including hard and soft sediments and areas with submerged macrophytes. The system was chosen for its relative narrowness, easing instrumentation of the system, and because the data reported by (Knutsen et al., 2018) suggested the fjord would contain a mixture of the North Sea and Fjord ecotypes.

Tidal amplitude of the system is 0.5 m on average. The surface current may be outgoing even during rising tides in periods with high freshwater runoff from rivers and streams entering the fjord and mixing poorly with more saline waters deeper down. The surface salinity is roughly 8–12 PSU in the inner fjord and 15–18 PSU in the outer fjord, while waters below a depth of roughly 6 m have a relatively stable salinity around 30–32 PSU.

### 2.2 Instrumenting the fjord

In October 2016, 13 acoustic receivers (Vemco VR2W, www.innovasea.com) were deployed in the fjord (Figure 1). Six receivers were deployed pairwise at three different transects of the fjord to facilitate an overall survey of what part of the fjord the different fish preferred to reside in. Two receiver gates with three and four receivers, respectively, were deployed in the outer part of the fjord to track movements of tagged fish in and out of the fjord system. The maximum detection distance to a receiver at the outermost transect was 130 m. Data were downloaded from the receivers in May, September, and November 2017 and in June 2018.

From May to November 2017, the receiver setup was expanded to an array covering the entire fjord when an additional 42 receivers (Thelma TBR 700, www.thelmabiotel.com) were deployed in the fjord system for another study. This provided more detailed position estimates of the tagged fish that were still residing in the fjord during this period. The array was not set up to perform precise 3D position estimates, but the array data could be used to provide position average estimates.

All receivers included in the study were deployed by anchoring the receiver to the bottom. The receivers were kept afloat, c. 3 m below the surface by an 8” float. Receivers and floats were covered in antifouling paint to prevent sinking and reduced detection range due to biofouling.

### 2.3 Sampling and tagging

One hundred and four cod were caught in collaboration with a local fisher using fyke nets on various locations at depths ranging between 1 and 8 m throughout the fjord between 25 October and 17 November 2016. Immediately after capture, the fork length of each fish was measured with a precision of 1 cm and a small fin‐clip was taken and stored in Eppendorf tubes containing ethanol to enable genetic origin assignment. Only fish measuring above 33 cm in length were sampled and tagged in order to narrow the size distribution of the fish included in the study. Apart from size, no selection was made on which fish to include, except for one individual that was bleeding from a severe injury, probably incurred by a seal, and therefore discarded. The fish included in the study were tagged with a T‐bar tag (Hallprint TBA2, 30 × 2 mm) printed with a serial number, return address, and a reward notice, and transported to a holding facility while being kept in a livewell on the boat. The holding facility consisted of fine‐meshed nets attached to a pontoon, enabling the fish to reside at depths down to 4 m while waiting to be tagged.

After a mean holding period of 13.2 days (range: 3–34 days), the fish were tagged with acoustic transmitters after being anesthetized with clove oil until the opercular rate became slow and irregular (2–4 min). An experienced fish surgeon tagged the fish with 9 mm Thelma acoustic tags (ID‐LP9L tags, www.thelmabiotel.com, 24 mm length by 9 mm diameter, 4 g in air, 2.5 g in water, transmitting with 142 dB re 1 uPa at 1 m) through a small incision on the ventral surface of the peritoneal cavity. The tags transmitted a unique ID at a random interval between 30 and 90 s (mean: 60 s) and had an expected battery life of 18 months.

The incisions were closed with two absorbable sutures, and a small (≈0.05 g) muscle biopsy was obtained from the dorsal region of each individual and stored in ethanol for subsequent analysis of δ^13^C and δ^15^N values in the fish. Fish were then left to recover in 200‐L containers of fresh fjord water. The operation lasted between one and two minutes, and the recovery time was 2–5 min. All tagged fish recovered from the procedure and were subsequently transported back and released at each of their respective sites of capture. All procedures were carried out in accordance with permission no. 6037 issued by the Norwegian Food Safety Authority.

### 2.4 Muscle sample analysis

Muscle tissue samples from biopsies of the 50 fish that generated data on the array deployed between May and November 2017 were analyzed with regard to stable carbon and nitrogen isotope ratios (δ^13^C and δ^15^N). Muscle samples were dried in aluminum foil trays at 45°C for 2–3 days. After drying, the samples were crushed and duplicate samples of 0.38 ± 0.1 (*SD*) mg tissue were packed in tin (Sn) cups for stable isotope analysis. All samples were analyzed at Department of Bioscience, Center for Geomicrobiology, University of Aarhus, Aarhus Denmark. The samples were measured by means of Isotope Ratio Mass Spectrometry (IRMS) in combination with an Element Analyzer (EA) and an operational interface (Thermo Electron Corporation Flash EA 1112 series and Thermo Scientific Delta V Plus Isotope Ratio MS).

The δ^15^N and δ^13^C values were standardized using a Gelatine A (Gel‐A) standard with known isotopic values of δ^15^N = 5.4‰ and δ^13^C = −21.8‰. For each nine or ten muscle tissue samples, three or two internal 0.2–0.7 mg Gel‐A standards were analyzed. The standards were used to correct for daily offsets and drift by regressing the measured isotope value of the internal standards on run number and correcting all muscle samples using the slope and intercept of this relationship and the known isotopic values of the internal standards. Low sample size bias was also assessed using the standards. The mean of the duplicate samples was used in data analyses.

### 2.5 Genetic analysis

A total of 104 tissue samples from candidate cod sampled in Sandnesfjord were genotyped for the present study. Tissue samples were extracted for DNA using the E.Z.N.A MicroElute Genomic DNA Kit (Omega BioTek), following the manufacturer's instructions for tissue samples with only one minor modification: the last elution buffer step being done twice through the same filter (50 µl was eluted). Genomic DNA from juvenile and spawning cod was extracted from a small piece of the dorsal fin, using E.Z.N.A Tissue DNA kit (Omega Bio‐tek) following the protocol. DNA from every individual was quality‐verified and quantified with a NanoDrop instrument (NanoVue Plus, GE healthcare). Twenty‐seven SNPs were previously developed to segregate between “Fjord‐” and “North Sea” individuals, and there were genotyped on a MassARRAY platform (Sequenom Inc.) at the IMR laboratory in Bergen, Norway. Genetic assignment of individual cod to ecotype was computed using the GeneClass2 software (Piry et al., 2004), using previously sampled reference populations of “Fjord” and “North Sea” cod (see Jorde, Synnes, et al., 2018 for additional information). The Bayesian method of (Rannala & Mountain, 1997) was used where a score >80% is needed in order to classify each individual either as a North Sea ecotype or Fjord ecotype. Omission of scores lower than 80% (*n* = 24) and individuals that were genotyped at <20 SNPs (*n* = 3) from further analysis resulted in 77 individuals being assigned successfully enabling selection for acoustic tagging. Five individuals had escaped or were potentially predated from the holding facility in the meanwhile, ultimately leading to 72 individuals being tagged.

### 2.6 Data analysis

Tagged fish were considered to have left the fjord system if their last detection occurred on one of the receivers in the outer transect (Villegas‐Ríos et al., 2020). The time of departure from the fjord was defined as the time of the last detection in the outer transect, and any subsequent returns to the fjord system were defined as the time of the first detection back at the outer transect.

Position averages were calculated with the array data (deployment time May–November 2017) for a total of 50 tagged fish still generating data in the fjord during this time. The position average (in UTM coordinates) of a fish detected a number of times on, for example, receiver X1 and X2 during an i'th 30‐min period was acquired as follows (Simpfendorfer et al., 2002):

$${\text{Position}}_{i}=\frac{\left(\text{No}{\text{. detections}}_{\text{X1}}\times {\text{Coordinates}}_{\text{X1}}+\text{No}{\text{. detections}}_{\text{X2}}\times {\text{Coordinates}}_{\text{X2}}\ldots \right)}{\left(\text{No}{\text{. detections}}_{\text{X1}}+\text{No}\text{. detection}s_{\text{X2}}\ldots \right)}.$$

The distance to the fjord outlet was calculated for each position average and used in the further analysis. Estimated 95% home range of each fish was calculated based on the position averages using the minimum convex polygon from the R package adehabitat (Calenge, 2006). Mean distance to the Skagerrak and the 95% home range size were entered into general linear models as dependent variables along with fish ecotype (North Sea or Fjord) and fish length. δ^13^C and δ^15^N values of the tagged fish were entered as response variables to investigate whether behavior (home range size and mean distance to the Skagerrak), fish size, or ecotype could explain any differences in δ^13^C and δ^15^N values in the fish. Insignificant covariates were dropped from the model. Collinearity between the entered variables was tested with the VIF function from the car package in R (Fox & Weisberg, 2019). Isotopic niche widths were calculated based on residuals from a GLM with isotope value (δ^13^C or δ^15^N) as response variable and distance to Skagerrak as predictor. Convex hull and standard ellipses were calculated and plotted using the package SIBER v2.1.4 in R (Jackson et al., 2011).

Home range sizes were analyzed with a general linear model with log‐transformed home range sizes entered as response variable and ecotype and fish length entered as dependent variables.

A gamma‐distributed linear mixed effects model from the R package glmmTMB (Brooks et al., 2017) with a log link was used to investigate whether fish of different ecotypes preferred residency closer or farther from the Skagerrak than each other throughout the period when the array was deployed. Distance to the fjord outlet was entered as response variable and fish origin (North Sea vs. Fjord), fish size, and time since 1 May 2017 were entered as dependent variables.

## 3 RESULTS

### 3.1 Tagged fish

Seventy‐two cod were sampled, tagged, and subsequently released at their capture location in the Sandnesfjord (Tables 1 and A1). Thirty‐one of these (43%) were North Sea ecotypes, and 41 individuals (57%) were Fjord ecotypes. The fish were caught and released on locations with a mean distance to the Skagerrak of 3.42 km (North Sea fish) and 3.68 km (Fjord fish).

**TABLE 1:** Summary data on tagged Atlantic cod *Gadus morhua* of the two ecotypes. Fish lengths were obtained at the time of capture in autumn 2016. Columns to the left show the data for all tagged fish while columns to the right show the data for the subset of individuals still alive and present in May–November 2017 when the array was deployed. Detailed information is reported in Table A1

Ecotype	All tagged individuals		Fish present in May–November 2017	
	*N*	Mean length (cm)	*N*	Mean length (cm)
NS	31	44.4 (range: 36–63)	15	44.1 (range: 36–60)
FJ	41	50.0 (range: 33–70)	35	50.4 (range: 33–70)
All	72	47.6	50	48.5

### 3.2 Movement ecology

**FIGURE 2:**
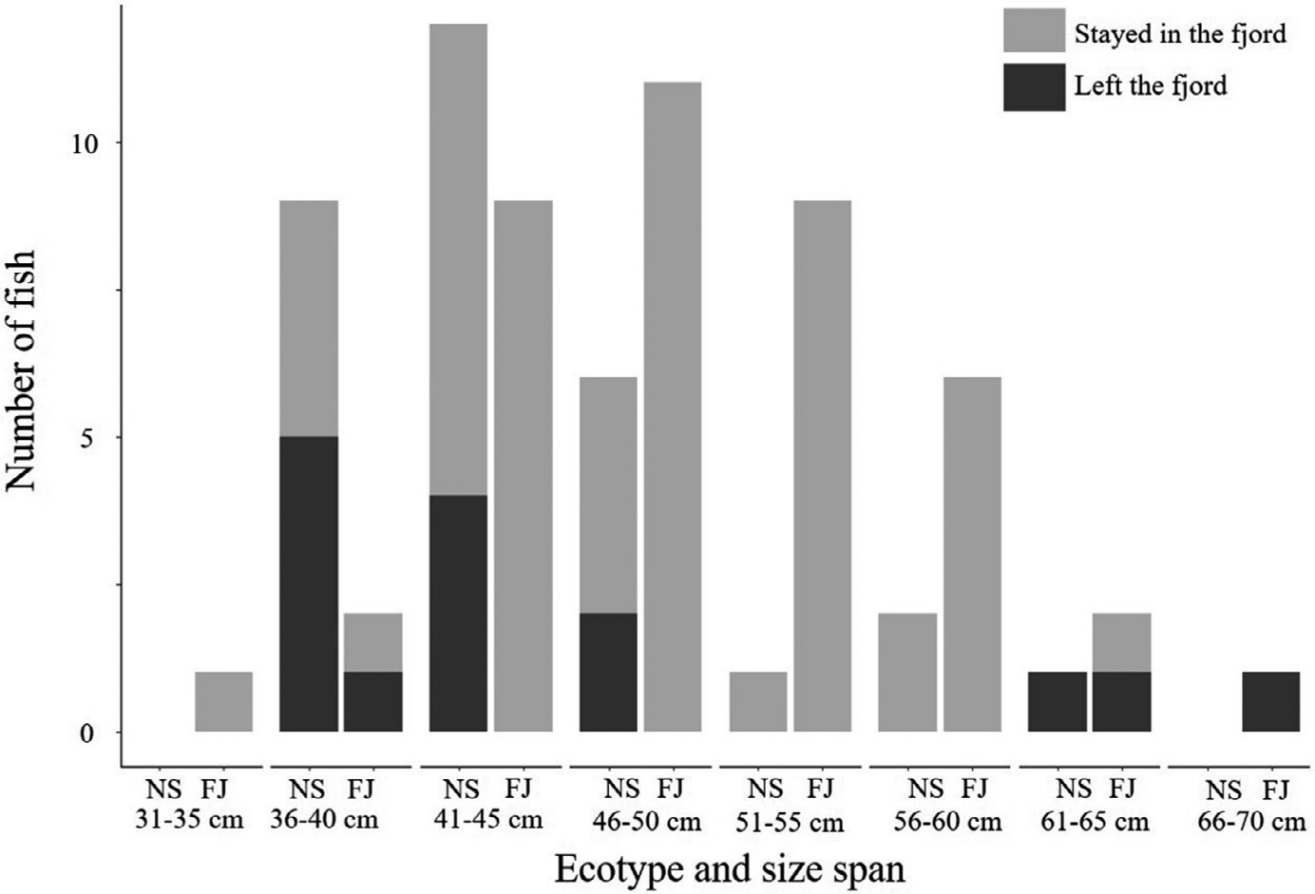
Size distribution and ecotype (NS = North Sea, FJ = Fjord) of tagged Atlantic cod *Gadus morhua*. Dark gray shading denotes fish that were detected as having left the fjord during the study period

During the entire study period, 12 North Sea ecotype (39% of tagged individuals assigned to the North Sea ecotype) and three Fjord ecotype (seven percent of tagged individuals assigned to the Fjord ecotype) left the fjord without returning (Figure 2). In addition, one North Sea ecotype individual left the fjord in December 2016 and returned again in April 2017, meaning that a total of 42% of tagged North Sea ecotype individuals left the fjord permanently or for a prolonged period of time (months) during the study. Of the fish that left the fjord, nine North Sea individuals and two Fjord individuals did so during the first winter (December 2016–February 2017), one North Sea fish did so during spring 2017 (March), two North Sea and one Fjord fish did so during summer 2017 (June–August), and one North Sea fish did so in winter 2018 (February). In addition to the fish that left the fjord permanently or for a long period of time, six individuals left the fjord for short periods of time (<2 days) during the study period. The fish that left the fjord for short periods of time were generally residing in the outer parts of the fjord system. No fish were detected on the outermost receiver transect without prior detection on the secondary transect located roughly 500 m further into the fjord, and no returning fish were detected on the secondary transect without prior detection at the outermost transect. The efficiency of the receiver gates at the fjord entrance was therefore considered high.

The two‐km long, inner section of the fjord was only rarely used by the tagged fish. Nine fish were detected in this fjord section for short periods of time (<2 days) during the study period while one North Sea individual resided there throughout the study period. The remaining fish that did not leave the fjord system spent all of their time within the seven km of fjord stretching from the receivers at the inner fjord section to the outer receiver line at the Skagerrak boundary.

Fifty of the 72 individuals that were tagged and assigned were still present in the fjord during May–November 2017 when the expanded array was operational. Twelve tagged fish had left before the array was deployed, meaning a total of 10 tagged fish had either died or shed the tag into an undetectable place, left the system undetected, or been removed from the system by fisheries.

**FIGURE 3:**
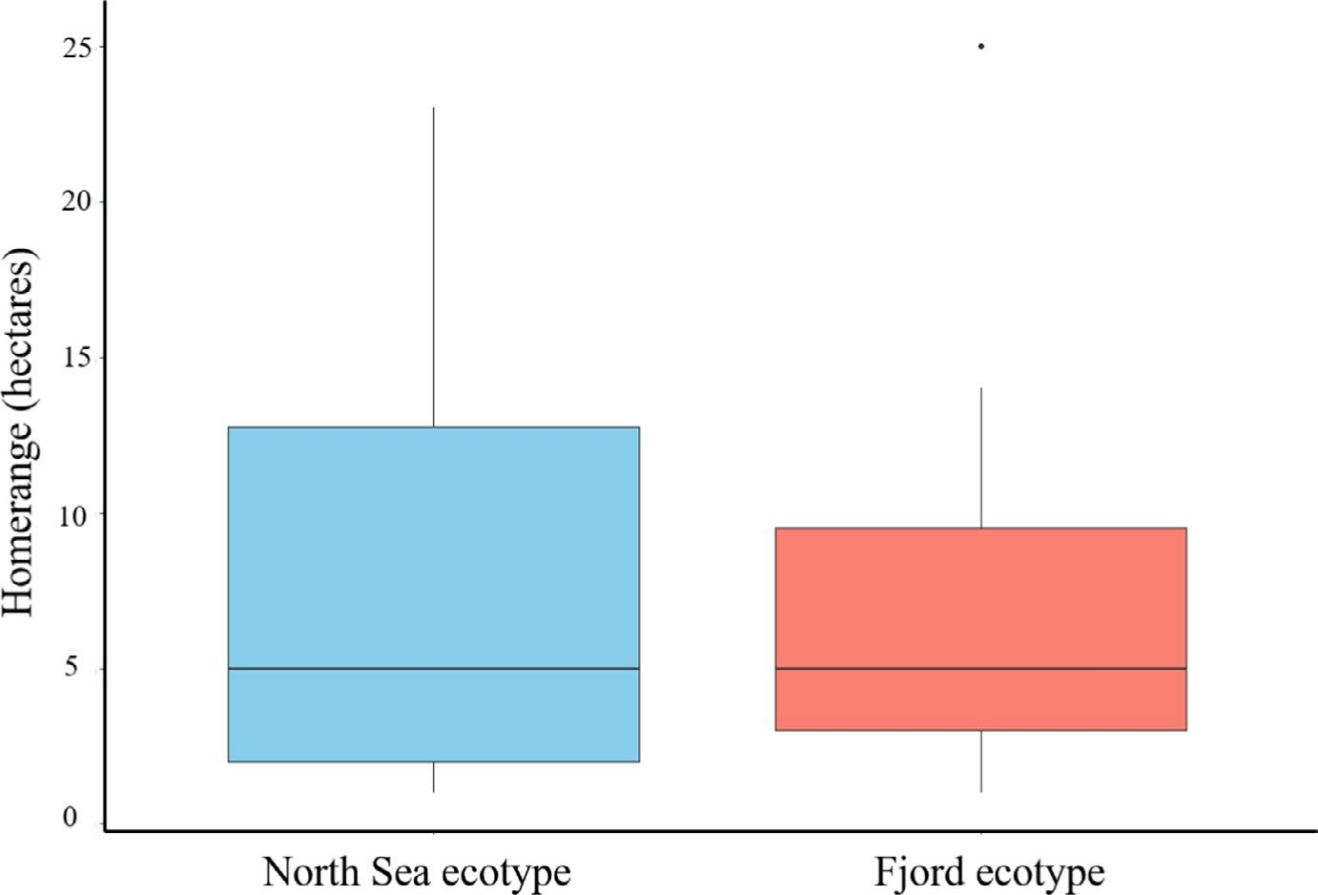
Boxplot of 95% home range area of tagged Atlantic cod *Gadus Morhua* with black horizontal lines representing median values, boxes representing the interquartile range of values from the 25th to the 75th percentile, vertical lines extending to 1.5 times the interquartile range and points representing outliers

The majority of the 50 fish present during the array deployment were sedentary most of the time and mostly detected on the same 2–3 receivers. Some individuals did perform excursions throughout larger areas in the fjord. As a consequence, home range sizes varied from 1 to 25 hectares (mean: 7.1 ha, *SD*: 5.9 ha, median: 5.0 ha), with no clear difference between the two ecotypes (Figure 3).

The general linear model output had no significant effect of ecotype (*p* = 0.660) or fish length (*p* = 0.637) on home range sizes of the fish (Table 2). Also, the interaction between ecotype and length was not significant (*p* = 0.695). Adjusted *R* ^2^ of the model was 0.05.

**TABLE 2:** Output from the general linear model of the effect of ecotype and fish length and the interaction between the two on home range size

	Value	*SE*	*T*‐value	*p*
Intercept	2.449	1.581	1.546	0.129
Ecotype	−0.845	1.914	−0.443	0.660
Length	−0.016	0.034	−0.475	0.637
Type × length	0.016	0.041	0.394	0.695

**FIGURE 4:**
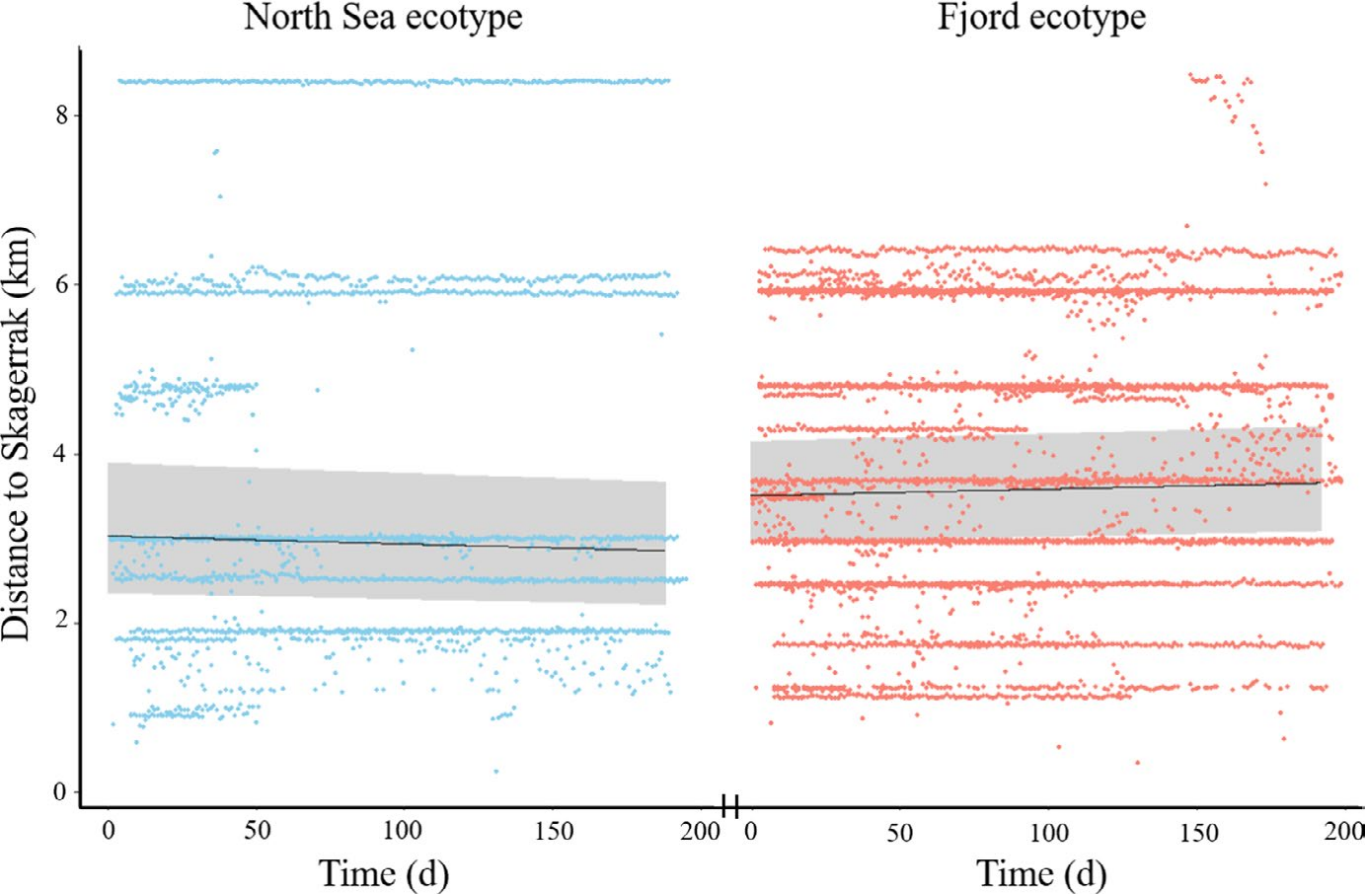
Distance to the Skagerrak for daily position averages for the two ecotypes of Atlantic cod *Gadus morhua* from May to November 2017 (dots) and output from mixed effects model of distance to the Skagerrak for the two ecotypes during May–November 2017 (black lines). Shaded areas represent 95% confidence intervals of the model

The generalized linear mixed modeling of residence distance from the sea found no significant difference between the ecotypes and detected no overall movement toward or away from the Skagerrak over time (Table 3). The results suggested individuals of both the North Sea and Fjord ecotypes were scattered across the fjord system with a small but insignificant skew of North Sea fish closer to the Skagerrak than individuals of the Fjord ecotype (Figure 4).

**TABLE 3:** Output from the mixed effects model of distance to the Skagerrak with time for the two ecotypes during May–November 2017

	Value	*SE*	*z*‐value	*p*
Intercept	1.109	0.1276	8.695	<0.0001
Ecotype	0.1534	0.1525	1.006	0.315
Time	−0.0031	0.0004	−6.891	<0.0001
Type × time	0.0005	0.0001	9.403	<0.0001

### 3.3 Isotopic niche

General linear models were used to investigate whether behavior (95% home range and distance to Skagerrak), ecotype (North Sea or Fjord), and fish length affected the δ^13^C and δ^15^N values of the fish and thus their trophic niche. There was no correlation between δ^15^N and δ^13^C values from North Sea (Pearson, *r* = 0.22, *p* = 0.23) or Fjord cod (Pearson, *r* = −0.003, *p* = 0.98); hence, the analysis was performed on the actual untransformed isotope values.

**FIGURE 5:**
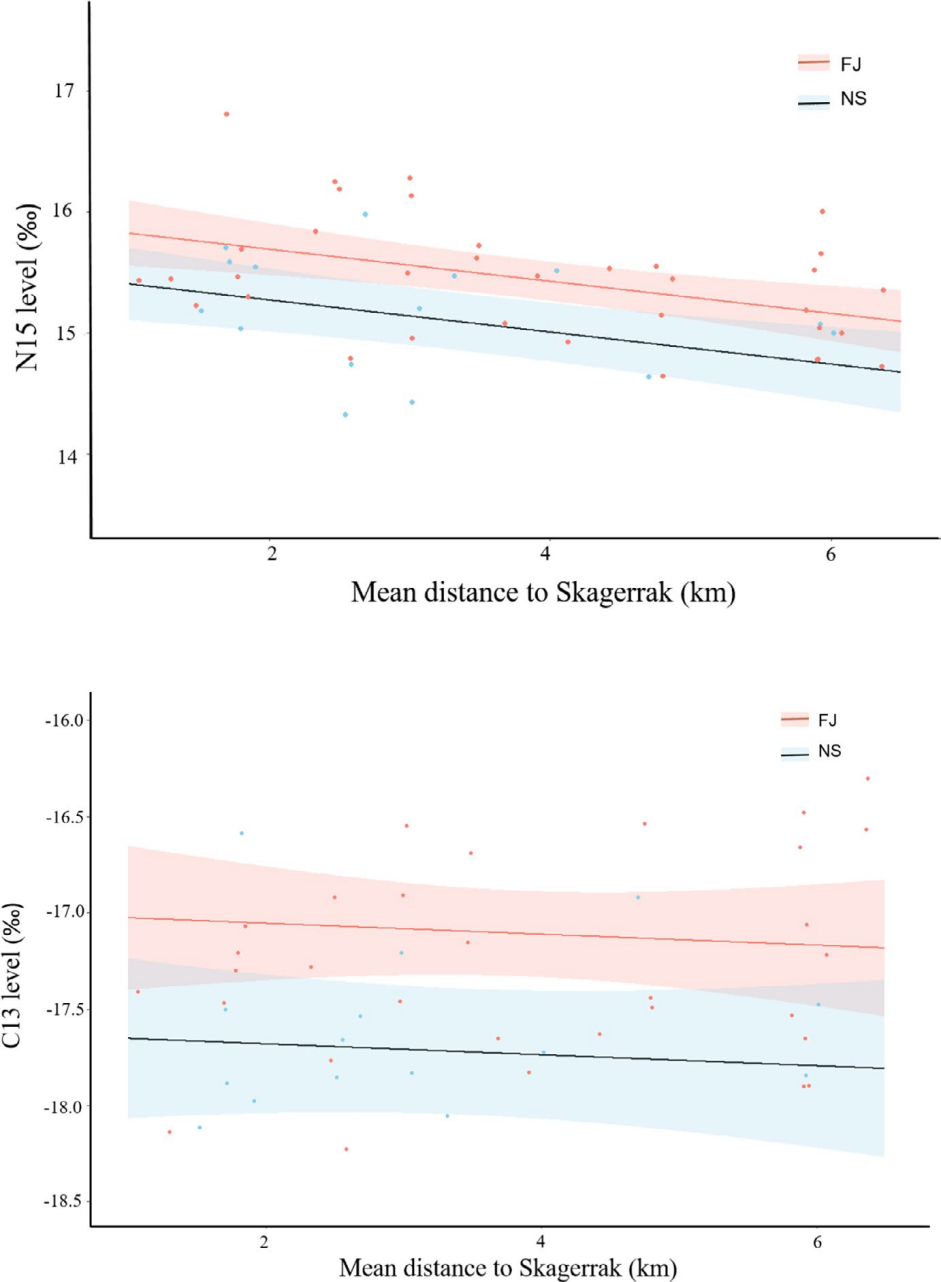
Linear model of δ^15^N (top panel) and δ^13^C (lower panel) in Atlantic cod *Gadus morhua* of the two ecotypes related to the mean residence distance from the Skagerrak in May–November 2017. Shaded areas represent 95% confidence intervals of the model, and points represent isotope levels and mean residence distances from Skagerrak for individual fish

The δ^15^N value (*p* = 0.010) and distance to the Skagerrak (*p* = 0.001) were significantly different between cod ecotypes (Figure 5, Table 4). VIF score of the two variables (1.052) suggested no problems with collinearity between them (24). The δ^13^C value was significantly different between cod ecotype (*p* = 0.003, Figure 5, Table 4). The interaction effect between ecotype and distance was not significant for either δ^15^N (*p* = 0.647) or δ^13^C (*p* = 0.121) and were therefore dropped from the final models. *R* ^2^ values of the final models were 0.248 for δ^15^N and 0.133 for δ^13^C.

**TABLE 4:** Output from the general linear models of the effect of ecotype, distance to the Skagerrak, fish length, home range size, and the interaction between ecotype and distance to the Skagerrak on stable isotope values

	Nitrogen				Carbon			
	Value	*SE*	*T*‐value	*p*	Value	*SE*	*T*‐value	*p*
Ecotype	0.389	0.146	2.663	0.010	0.626	0.198	3.160	0.003
Distance	−0.135	0.038	−3.566	0.001	−0.038	0.055	−0.696	0.490
Eco × Dist.	0.032	0.080	0.408	0.647	0.173	0.110	1.579	0.121
Length	−0.009	0.010	−0.859	0.592	−0.013	0.014	−0.954	0.351
Home range	0.011	0.013	0.911	0.505	0.004	0.017	0.245	0.539

**FIGURE 6:**
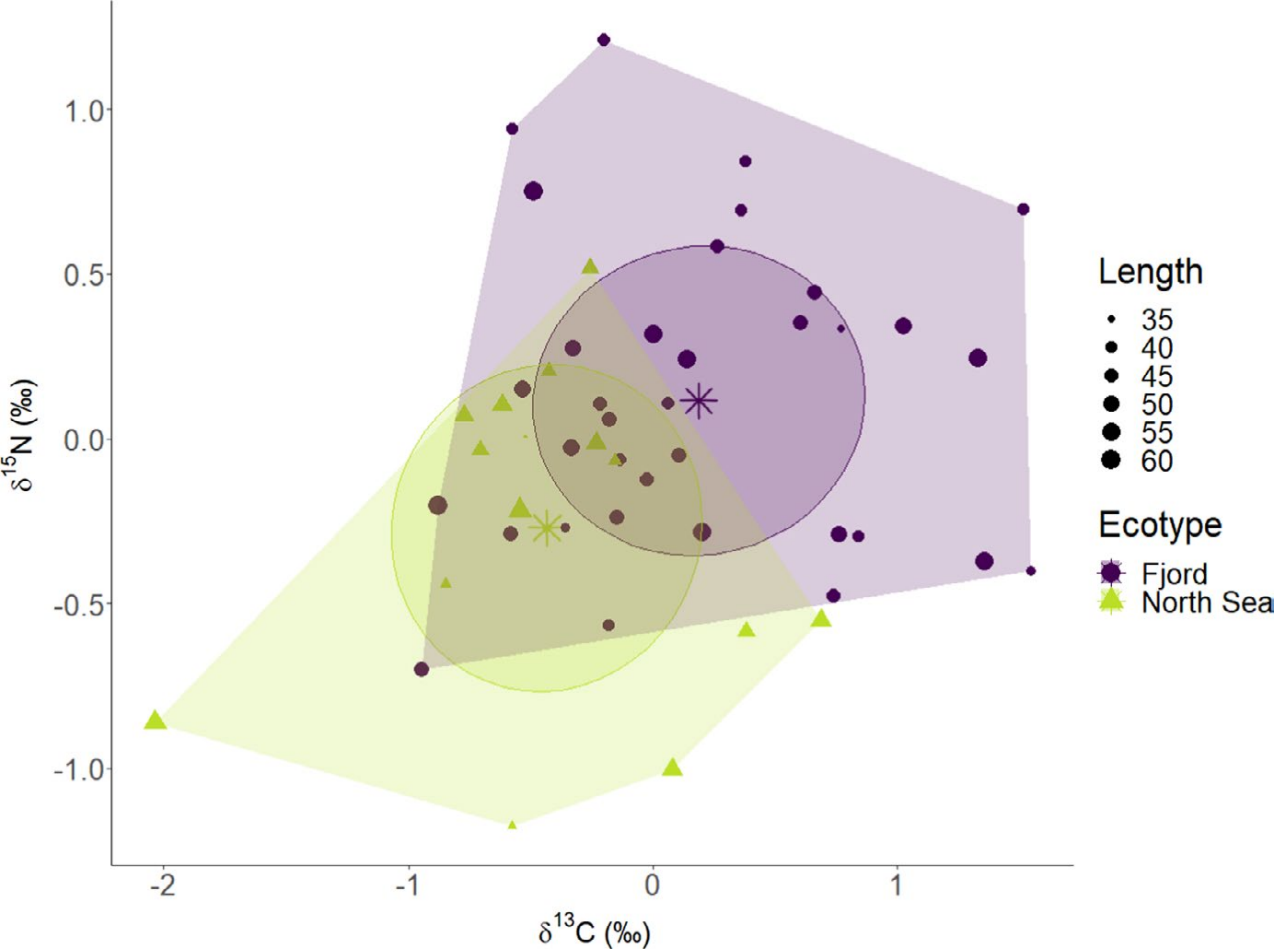
Biplot of residuals from the linear model of isotope values versus distance to Skagerrak. The convex hulls (polygons) and standard ellipse area (SEA) (ellipses) are plotted. Bivariate means for each ecotype are shown with stars and fish standard length in cm is indicated by symbol size

Residuals from the linear model of isotope values versus distance were plotted as a biplot (Figure 6). This showed a clear distinction between the two ecotypes, the Fjord ecotype having higher average residual values for both nitrogen and carbon than the North Sea ecotype. There was an overlap in isotope niche space among between the two ecotypes, but the isotopic niche width was considerably larger in the combined data than in either of the two ecotypes. The isotopic niche widths as expressed by sample size‐corrected standard ellipse areas (SEAc) were similar among ecotypes (North Sea = 1.00 ‰^2^ and Fjord = 0.99‰^2^) despite the indications of different feeding ecologies. The overlap in sample size‐corrected standard ellipse area between the two ecotypes was 0.28‰^2^, which is less than 1/3 of the individual ecotype standard ellipse areas, and the sample size‐corrected standard ellipse area of the combined dataset consequently increased to 1.11‰^2^. The isotopic niche width expressed as the convex hull areas were (TA) 2.35‰^2^ for the North Sea ecotype and 3.49‰^2^ for the Fjord ecotype. Treating the cod as one group yields a convex hull areas of 5.33‰^2^ or between 1.53 and 2.27 times the sizes of the individual trophic niche widths.

## 4 DISCUSSION

Our results document that sympatric, coexisting Atlantic cod ecotypes exhibit divergent migratory behaviors and feeding ecology. The North Sea ecotype was more likely to leave the fjord system compared to local Fjord cod and had significantly lower values of stable δ^13^C and δ^15^N isotopes. Given the potential importance of cod as a top predator, differences in fjord residence and trophic ecology may have an important effect on the overall structure and functioning of temperate coastal ecosystems.

In total, 43% of cod tagged with acoustic transmitters belonged to be North Sea ecotype, while the remaining 57% belonged to the Fjord ecotype. The genetic origin analysis has some uncertainty (5%) in assigning fjord individuals correctly (Jorde, Synnes, et al., 2018) and a few individuals might have been misclassified in our data. Despite any uncertainty, these results document that both ecotypes coexist at the same time in Skagerrak fjords, possibly with more or less asynchronously fluctuations in abundance among years (e.g., Knutsen et al., 2018). As a consequence, abundance of cod in the fjords may fluctuate regardless of local management initiatives.

The two cod ecotypes displayed divergent migratory behavior: 42% (*N* = 13) of the North Sea cod left the fjord permanently (*N* = 12) or for a prolonged period of time (4 months, *N* = 1) while only 7% (*N* = 3) of the Fjord cod left the fjord permanently. The size at maturity (50% probability) for broad samples of cod varied between 35 and 63 cm in different fjord systems along the Skagerrak coast in Olsen et al. (2004), while mean length of spawners of the FJ ecotype was 40 cm in Olsen et al. (2008). Estimates of the same for NS ecotype individuals inhabiting the coastal Skagerrak fjord systems are not available to date. Recent estimates of age and size at maturity (50% probability) in cod from the North Sea proper was 2.7 years and 44.6 cm, and 2.8 years and 46.1 cm for male and female cod, respectively (Marty et al., 2014). If assuming that the NS ecotype found in coastal Skagerrak is indeed similar to cod from North Sea proper in this regard, this would imply that fish in the size window of emigrating cod (36–70 cm at the time of tagging) observed in the present study might have been mature individuals and that their departure from the fjord might have been related to spawning. Natal homing has been extensively documented on Atlantic cod (André et al., 2016; Svedäng et al., 2010), and we hypothesize that North Sea cod left the fjord in order to return to their natal spawning grounds. This is supported by the time of departure from the fjord, as 10 of the North Sea individuals and two of the Fjord individuals left during spawning season in winter, similar to the migration timing observed in (Svedäng et al., 2007). Other population structuring mechanisms besides natal homing persist in cod populations (André et al., 2016; Skjaeraasen et al., 2011; Svedäng et al., 2010) where straying may be one of the most significant ones (Kovach et al., 2010; Svedäng et al., 2010). The Fjord fish that left during the spawning season could have done so to spawn in neighboring fjords or potentially strayed elsewhere although the mechanisms behind straying in cod are still poorly understood (Robichaud & Rose, 2001). The 58% (*N* = 18) of North Sea cod that stayed in the fjord or potentially died in it during the study period might be termed strayers if they spawned in the fjord. Barth et al. (2019) observed a similar and stable degree of co‐occurrence of ecotypes in a neighboring fjord system. Further exploration of fine‐scale behavior might uncover whether long‐term residents of the NS ecotype spawn separately from FJ individuals within fjord systems along the Skagerrak coast.

Two North Sea individuals and one Fjord individual left the fjord during summer without returning during the study period. These summer migrations were unlikely related to spawning, but could be a consequence of home ranges extending outside the fjord, a movement to avoid high summer temperatures within the fjord or a consequence of predation events. As observed in our study and with greater detail on cod in an adjacent fjord system by (Villegas‐Ríos et al., 2017), cod individuals may exhibit a wide variety in home range size from almost completely sedentary to highly migratory. The fish that left during summer could have simply died while residing outside the fjord. It has previously been documented that Atlantic cod avoid extreme temperature ranges either by vertical positioning in the water column (Espeland et al., 2010; Righton et al., 2010) or by selecting habitat based on bottom substrate (Freitas et al., 2016). This is important because suboptimal temperature may have various effect on physiological state of the fish and through that may have negative effect on different fitness‐related components, for example, growth (Righton et al., 2010). Therefore, it may be that conditions outside the fjord, in deeper and colder waters, may be more suitable for some individuals during the warmer months. Finally, the fish could have been predated inside the fjord by seals that subsequently left the fjord toward the seal colony located outside the fjord and array with tags still in their belly.

Individuals from both ecotypes were present throughout most of the fjord system and displayed similar home range sizes. The Fjord cod resemble cod from the southern Kattegat and western Baltic Sea (Barth et al., 2017) that are adapted to lower salinities (Larsen et al., 2012) and a relatively higher distribution of Fjord cod could have been expected deeper in the fjord where salinities are lower. The capture and release location of the 10 North Sea fish that left the fjord during the spawning season was 3.58 km as opposed to 3.42 km in all the assigned North Sea individuals, suggesting that the North Sea fish that left the system before the array was deployed had been similarly distributed throughout the fjord compared to the individuals that stayed.

Overall home range patterns for the fish included in the present study resembled those observed with greater detail by (Villegas‐Ríos et al., 2017) although generally smaller in the present study. This is likely a methodologically driven difference, as position averages as used in the present study will draw the fish positions toward the center of detection likelihood and thus underestimate the home range size. Position averaging delivers too coarse positions to enable unbiased determination of dead fish in the system, and some of the sedentary individuals in the present study could be dead individuals. The natural mortality for larger cod in neighboring fjords is, however, very low as a contrary to the annual fishery induced mortality of 50% or more, accounting for up to nearly 100% of the total mortality in large cod in coastal areas (Fernández‐Chacón et al., 2017; Olsen & Moland, 2011). Tag shedding also acts as a potential error source, although considered to be a small one. Twenty cod recaptured in a neighboring fjord after being acoustically tagged by the same fish surgeon as in the present study, all carried the tag when recaptured later on (E. Moland Olsen, pers. comm.). In spite of these sources of uncertainty, home range sizes estimated from position averages should still reveal differences between the ecotypes on a group level. Although highly variable between individuals, results from the present study suggested no such differences in home range sizes were present between the North Sea and Fjord ecotypes.

Differences in isotopic niche were observed between the two ecotypes. Cod from the North Sea exhibited lower δ^13^C and δ^15^N values compared to Fjord cod; for both ecotypes, the δ^15^N values were related to the distance to the outlet of the fjord. These results suggest that the diet composition of the North Sea ecotype differs from that of the Fjord ecotype.

Cod in the southern Norwegian fjords are omnivorous, and in the present size range, they primarily feed on a mixture of fish, decapods, polychaetes, and gastropods (Salvanes et al., 2004). The proportions of these prey groups vary by season, similar to what is seen in other populations (Grønkjær et al., 2020; Link et al., 2009). While the fish ingested may be both benthic and pelagic, the decapods, polychaetes, and gastropods are primarily benthic predators, deposit feeders, or scavengers forming part of a benthic food web. Pelagic and benthic food webs can be distinguished based on the δ^13^C values as benthic food webs are characterized by higher δ^13^C values than their pelagic counterparts (Telsnig et al., 2019). Unfortunately, there are no prey isotope data from the fjords investigated in this study, but the pattern has been documented in a comparable fjord system in Northern Norway, where the benthic community showed higher δ^13^C (Shrimps δ^13^C = −17.5‰; Large crustaceans δ^13^C = −20.0‰; Predatory benthos δ^13^C = −17.9) compared to pelagic prey (Herring δ^13^C = −21.3; Krill δ^13^C = −22.4). An explanation for the ecotype specific isotopic values, which is consistent with known diet composition (Link et al., 2009; Grønkjær et al., 2020; Mattson, 1990) and isotopic values of prey (Giraldo et al., 2017; Telsnig et al., 2019), could therefore be an increased proportion of benthic scavengers and deposit feeders compared to pelagic organisms in the diet of the Fjord ecotype. The increased reliance on benthic food sources may be an adaptation to the shallow coastal and fjord habitats, where the production of benthic prey is higher than in offshore habitats. In more offshore populations and locations, there is a tendency toward increasing proportions of fish in the diet compared to coastal locations (Dalpadado & Bogstad, 2004; Hedeholm et al., 2017; Pálsson & Björnsson, 2011). This may be driven by increased availability of a wider range of pelagic fish species (e.g., herring, sand lance) and the effect of occupancy is augmented by the generally larger size of offshore cod (Berg & Albert, 2003; Roff, 1988), which allow them to prey more efficiently on larger fish prey. In contrast, for the coastal populations, higher biomasses of benthic prey in the shallower waters provide these cod with improved benthic feeding conditions (Mattson, 1990). The higher δ^15^N in the fjord ecotype suggests that a large proportion of their diet consists of benthic scavengers and predators which have high δ^15^N values (Giraldo et al., 2017; Tamelander et al., 2006) compared to benthic suspension feeders and grazers. The importance of brachyuran (true crabs) and anomuran decapods in the diet of cod in the area supports this (Hop et al., 1992). The decrease of δ^15^N toward the mouth of the fjord is consistent with anthropogenic eutrophication within the fjord and mixing with less eutrophied coastal water as seen in other systems (Cabana & Rasmussen, 1996; Kristensen et al., 2019). This leads to a decreasing δ^15^N baseline from the head to the mouth of the fjord, which is reflected in the consumers.

This is the first study to document dietary differences among genetically divergent ecotypes of cod inhabiting the same environment and subsequently study the behavior of individual fish. The results indicate adaptation to local prey types in the local Fjord ecotype and lack of adaptation within a month‐to‐year timescale in the alien North Sea ecotype. Previous studies of reared cod have shown differences in behavior of individuals from genetically different populations and suggested that higher growth of cod from the Northern coast of Norway was due to more active feeding strategy on pelagic prey compared to the Southern origin cod (Salvanes et al., 2004). Our study takes this down to the level of co‐occurring ecotypes. Also, (Knutsen et al., 2018) and (Jørgensen et al., 2020) found growth differences between the two ecotypes, where juveniles of the North Sea ecotype display faster growth than the local Fjord type. The present study and the study by Salvanes et al. (2004) suggest that observed growth differences may be driven by differences in feeding ecology and be maintained throughout the life of the cod.

The clear differences in diets, behavior, and growth of the Fjord and North Sea ecotype cod suggest that the two ecotypes will have distinct effects on the fjord ecosystem. Depending on the ratio between ecotypes within the fjord, which is subject to change over time (Knutsen et al., 2018), different prey items will be under dynamic predatory pressure, which may have an effect on the abundance and composition of different elements in the food web. Similarly, the abundance of the two ecotypes may be driven by availability of the relevant prey types (pelagic vs. benthic), and hence, the occurrence of two ecotypes with distinct prey requirements may offer resilience in terms of cod survival. The distinct prey requirements are seen in the low degree of overlap in isotopic niche, which allow cod ecotypes to coexist and together utilize a broader dietary niche than if only one of the ecotypes had been present. Therefore, the loss of one ecotype fish may have significant ecological effects on the overall functioning of the ecosystem. Our results highlight the importance of ensuring sustainable population developments in interconnected populations in order to maintain marine ecosystem functioning and resilience to environmental change.

## CONFLICT OF INTEREST

All authors declare to have no conflicts of interest.

## AUTHOR CONTRIBUTIONS

**Martin Lykke Kristensen:** Conceptualization (supporting); Data curation (lead); Formal analysis (lead); Investigation (equal); Methodology (equal); Writing‐original draft (lead); Writing‐review & editing (lead). **Esben Moland Olsen:** Conceptualization (equal); Data curation (equal); Formal analysis (supporting); Funding acquisition (supporting); Investigation (equal); Methodology (equal); Project administration (equal); Supervision (equal); Writing‐original draft (equal); Writing‐review & editing (supporting). **Even Moland:** Conceptualization (supporting); Data curation (equal); Formal analysis (supporting); Funding acquisition (supporting); Investigation (equal); Methodology (equal); Supervision (equal); Writing‐original draft (equal); Writing‐review & editing (supporting). **Halvor Knutsen:** Conceptualization (equal); Data curation (equal); Formal analysis (equal); Funding acquisition (lead); Investigation (equal); Methodology (equal); Project administration (lead); Resources (equal); Writing‐original draft (equal); Writing‐review & editing (supporting). **Peter Grønkjær:** Conceptualization (equal); Data curation (equal); Formal analysis (equal); Funding acquisition (equal); Investigation (equal); Methodology (equal); Supervision (equal); Writing‐original draft (equal); Writing‐review & editing (equal). **Anders Koed:** Data curation (supporting); Funding acquisition (supporting); Methodology (supporting); Project administration (supporting); Resources (equal); Supervision (supporting); Writing‐original draft (equal); Writing‐review & editing (supporting). **Kristi Källo:** Formal analysis (supporting); Methodology (supporting); Writing‐original draft (equal); Writing‐review & editing (supporting). **Kim Aarestrup:** Conceptualization (lead); Funding acquisition (equal); Methodology (equal); Project administration (equal); Supervision (equal); Writing‐original draft (equal); Writing‐review & editing (supporting).

### OPEN RESEARCH BADGES

This article has earned an Open Data, for making publicly available the digitally‐shareable data necessary to reproduce the reported results. The data is available at https://doi.org/10.5061/dryad.5hqbzkh63.

## Data Availability

Fish and tagging information is provided in the appendices. All data can be downloaded from the Dryad repository at https://doi.org/10.5061/dryad.5hqbzkh63.

## 1

**TABLE A1:** Fish tagging, movement and stable isotope data

Tag ID	Nitrogen	Carbon	Fishtype (1 = NS. 2 = FJ)	Length	Homerange	Distance to Skagerrak
1917	15.15	−17.44	2	42	8	4.8
1919	16.19	−16.92	2	41	3	2.5
1921	16.13	−15.77	2	40	1	3.0
1922	14.78	−16.48	2	40	13	5.9
1924	15.47	−17.30	2	45	11	1.8
1925	15.69	−17.21	2	42	2	1.8
1927	15.62	−17.15	2	56	3	3.5
1928	15.51	−17.72	1	43	15	4.0
1929	15.72	−16.69	2	48	2	3.5
1930	14.78	−17.90	2	48	3	5.9
1931	15.53	−17.63	2	50	7	4.4
1932	14.79	−18.23	2	48	4	2.6
1933	15.18	−18.11	1	38	4	1.5
1935	14.64	−16.92	1	45	5	4.7
1936	16.81	−17.47	2	42	10	1.7
1937	15.35	−16.30	2	54	10	6.4
1938	14.92	−15.94	2	55	10	4.1
1939	15.22	−15.72	2	37	2	1.5
1940	15.52	−16.66	2	48	6	5.9
1941	15.00	−17.22	2	48	3	6.1
1942	15.45	−18.14	2	60	1	1.3
1943	15.46	−18.06	1	50	23	3.3
1944	15.65	−17.06	2	44	8	5.9
1946	14.43	−17.21	1	52	2	3.0
1947	14.99	−17.48	1	40	21	6.0
1949	14.72	−16.56	2	51	25	6.4
1950	16.25	−17.77	2	59	5	2.5
1951	15.08	−17.84	1	34	8	5.9
1952	16.01	−17.90	2	41	11	5.9
1953	15.19	−17.53	2	44	5	5.8
1954	15.58	−17.50	1	51	12	1.7
1955	14.32	−17.85	1	36	5	2.5
1957	14.96	−16.55	2	44	3	3.0
1964	15.70	−17.88	1	50	2	1.7
1966	15.45	−15.98	2	58	5	4.9
1972	13.90	−19.38	1	55	1	8.4
1973	15.04	−17.65	2	52	9	5.9
1975	15.49	−17.46	2	48	5	3.0
1977	15.84	−17.28	2	57	1	2.3
1978	16.28	−16.91	2	40	2	3.0
1979	15.30	−17.07	2	56	14	1.9
1980	15.03	−16.58	1	52	4	1.8
1981	15.08	−17.65	2	38	5	3.7
1982	15.99	−17.54	1	46	5	2.7
1985	15.44	−17.41	2	47	12	1.1
1986	15.21	−17.83	1	58	22	3.1
1987	15.55	−16.54	2	35	7	4.8
1988	15.54	−17.98	1	45	1	1.9
1989	15.47	−17.83	2	52	7	3.9
1991	14.64	−17.49	2	41	5	4.8
